# Impact of a Pharmacist-Driven Penicillin Allergy De-Labeling Service (PADLS) on Hospitalized Patients

**DOI:** 10.3390/pharmacy14010033

**Published:** 2026-02-11

**Authors:** Parker Kaleo, Natt Patimavirujh, Kristen Greene, Nicholas Piccicacco, Melissa O’Neal

**Affiliations:** 1 Department of Pharmacy, Tampa General Hospital, 1 Tampa General Cir, Tampa, FL 33606, USA; patimavn@sjchs.org (N.P.); kzeitler@tgh.org (K.G.); npiccicacco@tgh.org (N.P.); moneal@tgh.org (M.O.); 2 Department of Pharmacy, St. Joseph’s/Candler Health System, 5353 Reynolds St, Savannah, GA 31405, USA

**Keywords:** allergy de-labeling, allergy reconciliation, antimicrobial stewardship, direct de-labeling, direct oral challenge, penicillin allergy, pharmacist-driven intervention

## Abstract

Penicillin (PCN) allergies are frequently reported despite a true prevalence of less than 1%, leading to unnecessary avoidance of beta-lactams, broader antimicrobial use, and increased healthcare costs. Pharmacist-driven de-labeling programs offer a strategy to improve antimicrobial stewardship. This single-center, retrospective study evaluated hospitalized adults with a documented PCN allergy and screened by the pharmacist-driven penicillin allergy de-labeling service (PADLS) between 16 January and 26 June 2025. Patients were categorized into a screened cohort and a Full Allergy Reconciliation (FAR) cohort if interviewed using PEN-FAST. Eligible patients underwent direct oral challenge (DOC), penicillin skin testing (PST) plus DOC, or direct de-labeling based on PEN-FAST scoring. Sixty-three patients were screened, and 32 (50.8%) underwent full reconciliation. Among FAR patients, the median PEN-FAST score was 0, and 25 (78.1%) underwent DOC. De-labeling was successful in 28 FAR patients (87.5%). One patient (4%) experienced a mild reaction. Allergy field updates occurred in 69.8% of screened and 96.9% of FAR patients. Antibiotic optimization occurred in 12 FAR patients, saving 78 days of therapy. Estimated cost savings totaled $37,632. PADLS effectively and safely de-labeled PCN allergies, resulting in improved antimicrobial selection, and could generate cost savings, supporting broader implementation of pharmacist-led allergy stewardship programs.

## 1. Introduction

Beta-lactam antibiotics remain one of the cornerstone treatment options for bacterial infections and surgical prophylaxis. These antibiotics are the largest class of antimicrobials with high utilization given demonstrated safety and efficacy; however, documentation of allergies to beta-lactams may limit their use [1,2,3,4]. In the United States, penicillin (PCN) allergies are the most commonly reported drug allergy, with approximately 10% of patients reporting hypersensitivity reactions, while the true prevalence is <1% [1]. PCN allergies dissuade healthcare providers from choosing beta-lactams as an antimicrobial choice, leaning towards non-beta-lactams such as clindamycin or fluoroquinolones [4]. Thus, PCN allergies have been associated with treatment failures due to suboptimal antibiotic usage, the use of unnecessarily broad-spectrum antibiotics, antibiotics with higher risks of adverse events, an increased chance of developing a multi-drug-resistant organism (MDRO), and increased healthcare costs [5,6,7].

Several publications highlight scoring tools for antibiotic allergies to properly stratify risk [8,9,10,11]. The PEN-FAST scoring tool, validated in patients with a reported PCN allergy, considers reaction type, timing of the reaction, and administered treatment to predict the likelihood of a positive skin test upon re-exposure [8,9]. While directly challenging an allergy is the new gold standard for de-labeling, historically, patients often underwent PCN skin testing (PST) before a direct challenge out of an abundance of caution. However, the PALACE trial found that the use of direct oral PCN challenge alone is non-inferior to PST plus oral challenge in de-labeling patients with low-risk PEN-FAST scores (0–1) [12,13]. Due to this, de-labeling via direct challenge has become more common; with less invasive, widely available de-labeling, more patients are able to be de-labeled [14].

Despite the benefits of PCN allergy de-labeling, several barriers limit its implementation in the inpatient setting. These include time constraints, clear delineation of roles and responsibilities when performing allergy de-labeling, concerns for precipitating an allergic reaction, and availability of training or competency in de-labeling practices [15]. Although allergists have traditionally been consulted to perform PCN allergy evaluations, it is not feasible to involve allergy specialists for the large volume of inpatients with low-risk PCN allergies. This gap has prompted the development of pharmacist-led initiatives; however, few programs empower pharmacists to perform direct de-labeling, direct oral challenges (DOC), and PST across all inpatient units and levels of acuity [16,17,18].

In January 2025, the Tampa General Hospital (TGH) antimicrobial stewardship pharmacist team established a pharmacist-driven PCN allergy de-labeling service (PADLS) with the capabilities to offer comprehensive de-labeling activities during hospitalization. Through PADLS, allergy assessments were proactively performed using PEN-FAST to determine appropriate allergy reconciliation steps for patients. These include de-labeling based on allergy history alone, a direct oral challenge, or a skin test followed by oral challenge. The goal of this study was to describe the ability of the PADLS program to assess and de-label PCN allergies.

## 2. Materials and Methods

### 2.1. Study Design

This was an Institutional Review Board (IRB)-approved, single-center, retrospective study of patients admitted to TGH with a PCN allergy and evaluated by PADLS from 16 January 2025–26 June 2025. This time period represents the first 6 months from PADLS program implementation. Included patients were 18 years of age or older, admitted to TGH, had a documented PCN allergy, were on systemic antibiotics, and were evaluated by PADLS. Patients were excluded if they could not tolerate oral medications or had gut dysmotility, had a history of drug induced liver disease, drug-induced thrombocytopenia, or severe cutaneous reactions (Stevens–Johnson syndrome, toxic epidermal necrolysis, drug reaction with eosinophilia and systemic symptoms, or acute generalized exanthematous pustulosis) related to beta-lactams, had a skin condition precluding PST, required PCN desensitization, or had recent use of immunosuppressive therapies or antihistamine agents that could mask a reaction for PST. Once patients were evaluated by PADLS to be appropriate for assessment, they were separated into two patient cohorts. The “Screened” cohort consisted of patients who met the inclusion criteria and were chart-reviewed. The “Full Allergy Reconciliation” (FAR) cohort included screened patients who were also interviewed and fully assessed using PEN-FAST by PADLS. Patients were not included in the FAR cohort if they or their providers declined participation or were unavailable for assessment. Owing to the retrospective design, some patients were not assessed for unspecified reasons; although they did not meet exclusion criteria, they were nevertheless not included in the FAR cohort.

### 2.2. PADLS Workflow

Patients were reviewed by PADLS to determine whether a full allergy history could be performed. This was dependent on patient availability for the interview and patient/provider consent for the interview. If the patient was not assessed, an attempt was made to update the allergy field in the electronic medical record (EMR) with best practice recommendations from expert guidance [1].

If the patient was assessed, they were interviewed by PADLS and included in the FAR cohort. The interview included detailed questions on the symptoms of their reaction, timing of reaction, any treatment or hospitalizations that may have been required, and agents that have been used since the reaction, including the same agent or structurally similar/dissimilar agents. Any patient who did not remember their allergic reaction was not deemed to have anaphylaxis, angioedema, or a severe cutaneous reaction and did not receive points on PEN-FAST. From this, a PEN-FAST score was calculated to stratify risk and appropriateness for de-labeling (Table 1).

Patients who reported intolerances (gastrointestinal upset, nausea, vomiting, and headaches), which are not immune-mediated processes, were offered direct de-labeling without a direct oral challenge (DOC) or PST. Patients were offered DOC if their PEN-FAST score was ≤2. Patients were offered a PST plus DOC if their PEN-FAST score was 3. Patients were not offered PST or DOC if they had a PEN-FAST score of ≥4 or had a severe cutaneous reaction.

PADLS requires interdisciplinary collaboration to be successful, as different members of the treatment team are responsible for portions of the allergy reconciliation process. Pharmacists were responsible for screening, conducting a thorough allergy history, communicating recommendations to providers, and ultimately verbally consenting the patient. Upon primary team agreement, the pharmacist then placed orders for DOC as well as rescue medications such as epinephrine, diphenhydramine, famotidine, and methylprednisolone. A DOC consisted of a 1-step challenge with 250 mg amoxicillin, or a 2-step challenge consisting of 25 mg amoxicillin followed by 250 mg amoxicillin, which was selected pursuant to the clinical judgment of the interviewing pharmacist in conjunction with patient preference. Medical and nursing staff were educated on the DOC process, and pharmacists were always available for guidance on timing, administration, or reaction treatments. As requested, pharmacy staff alerted the rapid response team to any ongoing DOC. If a reaction occurred, the reaction was evaluated by the attending physician. If a PST plus DOC was pursued, the pharmacist administered each step of the PST, with the results being read by an accredited physician. Previously published methods regarding skin prick and intradermal testing were followed [19]. After a 30 min monitoring period post-DOC, the pharmacy team confirmed tolerance with the patient and nursing staff. Results were communicated to the primary team, and the allergy was de-labeled from the EMR where appropriate. If desired by the patient, a wallet card indicating the date and result of the DOC or PST was provided. Regardless of whether the patient progressed to DOC or PST, pharmacists provided just-in-time education regarding PCN allergies, de-labeling, and cross-reactivity risk to patients and providers.

### 2.3. Outcomes

The primary outcome was to evaluate the number of patients successfully de-labeled by PADLS. Secondary outcomes included the number of patients able to have their PCN allergy field updated, DOC tolerability, the incidence of antibiotic adjustment after assessment, days of antibiotic therapy after adjustment, reasons why patients could not be assessed, and cost savings associated with de-labeling.

Successful de-labeling was determined at the time the patient was assessed or screened, not at the time of data collection. Allergy field updates included confirming the patient’s allergy after assessment, updating available agents that could be used based on cross-reactivity risk, and/or de-labeling the allergy. Antibiotic adjustments were only captured if they occurred after updating the allergy field. Antibiotic adjustments were included if the patient transitioned from a non-beta-lactam to PCN or cephalosporin, cephalosporin to PCN, or cephalosporin to a structurally dissimilar oral cephalosporin. Based on published cost-effectiveness data, each DOC followed by de-labeling was estimated to have a fixed cost savings of $1792 [20].

### 2.4. Data Analysis

All analyses were descriptive in nature, as the primary aim was to characterize the effectiveness and areas of improvement for PADLS without testing specific hypotheses. Continuous variables were reported as median [interquartile range (IQR)]. Categorical variables were summarized using frequencies and percentages.

## 3. Results

A total of 63 patients were evaluated by PADLS and included in this study. In the overall screened cohort, the majority of patients were Caucasian (n = 46, 73%) with a median age of 63 (IQR 53,73) years (Table 2). These patients were being treated for a wide variety of infections, predominantly pneumonia (n = 15, 23.8%), skin and soft tissue infection (SSTI) (n = 14, 22.2%), and urinary tract infection (UTI) (n = 13, 20.6%), but only 20 (31.7%) had an infectious diseases consultation. The median length of stay was 7 (3.5, 14) days, and the median duration from the start of antibiotic treatment until PADLS assessment was 3 (2, 7) days. The majority of patients were screened for PADLS while having a non-ICU floor status (n = 45, 71.4%).

**Figure 1:**
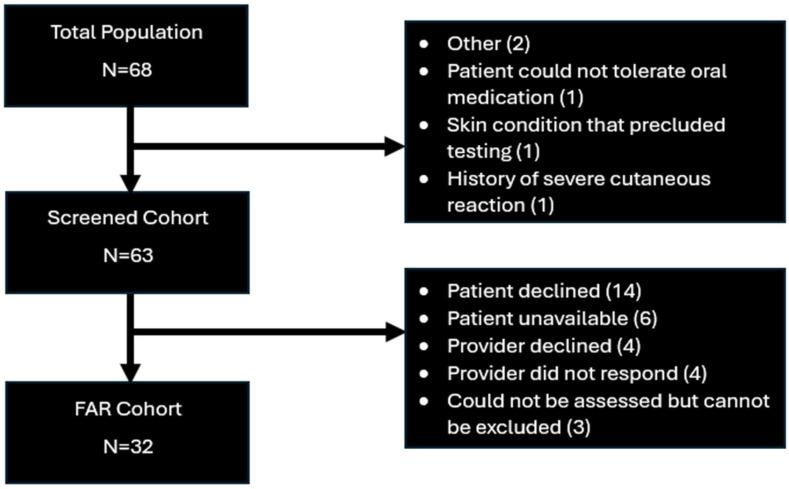
Patient cohorts.

Only 32 patients (50.8%) were included in the FAR patient cohort (Figure 1). Among patients not in the FAR cohort, the most common reasons were patient declination (n = 14, 45.2%) or provider declination (n = 8, 25.8%) of the assessment. For FAR patients, the median PEN-FAST score was 0 (0, 1) (Table 3). The majority of patients either had a history of a mild-moderate reaction (n = 14, 43.8%) to PCNs or an unknown reaction (n = 10, 31.3%). Twenty-one patients (65.6%) had a reaction more than 40 years ago. Twelve patients (37.4%) required an Emergency Department visit for their past reaction, but only one patient (3.1%) received epinephrine. Twenty-four patients (75%) received a one-step DOC, one patient (3.1%) received a two-step DOC, and seven patients (21.9%) were directly de-labeled without DOC or PST.

In terms of the primary outcome, the screened and FAR cohorts had 29 patients (46%) and 28 patients (87.5%) de-labeled, respectively (Table 4). There was one patient (4%) who did not tolerate DOC and required treatment with diphenhydramine and steroids. No patients required activation of the rapid response team. The number of patients who were able to have their allergy field updated was 44 (69.8%) in the screened cohort and 31 (96.9%) in the FAR cohort. The PADLS program was able to adjust antibiotics in 20 patients (31.7%), accounting for 127 days (23.6%) of the total duration of therapy in the screened group, and 12 patients (37.5%), accounting for 78 days (27.8%) of the total duration of therapy in the FAR group.

Estimated cost savings for the FAR cohort were $37,632 for 21 patients who underwent DOC and were successfully de-labeled. The PADLS program was carried out over ~48 h of direct pharmacist work, averaging $784 for every hour spent towards this initiative.

## 4. Discussion

The findings from this study highlight the real-world effectiveness of PADLS at de-labeling PCN allergies, updating allergy fields, and may be able to generate cost savings. We found that of eligible patients undergoing Full Allergy Reconciliation, ~90% were able to be prospectively de-labeled during their hospitalization. The majority of PADLS patients (75.0%) were successfully de-labeled via a one-step challenge, as described in prior studies, affirming the safety and ease of this practice in an inpatient setting [12]. However, as this is a fraction of the overall screened cohort (32/63, 50.7%), future protocols could be developed to ensure screened patients progress to full reconciliation. These findings are complementary to previously published reports on governance needed to feasibly de-label allergies in an acute care setting [13]. Patients in our study were also directly de-labeled pursuant to discussion (7/32, 21.9%), displaying a large proportion of non-immune-mediated reactions, not described in previous screening studies [12].

Building on this literature and current recommendations, several other pharmacist-led programs have advanced allergy stewardship. Vanderbilt University Medical Center successfully de-labeled low-risk patients through a 1-step DOC in the ICU setting. Mutua de Terrassa University Hospital was able to implement an algorithm to assess and de-label patients in the Emergency Department, and Oregon Health and Science University was able to directly de-label patients without any testing or challenge required [18,19,20]. In contrast to these programs, PADLS encompassed a broad hospital population and employed multiple de-labeling strategies (i.e., one-step, two-step, and direct de-labeling), rather than focusing on specific units, patient subgroups, or a single de-labeling strategy. While no PST was done during this study period, this is another de-labeling method able to be employed by PADLS. The ability to accomplish all of these de-labeling strategies across an inpatient population makes PADLS a comprehensive and progressive program. Despite this broad scope, optimization remains possible, including enhanced educational initiatives at both patient and provider levels to reduce hesitancy and expand safe de-labeling. Overall, PCN de-labeling rates and tolerability align with prior retrospective cohort studies, reinforcing that pharmacist-led programs can be powerful drivers of allergy stewardship and safely expanded in scope [12,13,18,19,20].

Among the four patients in the FAR cohort who were not de-labeled, all had PEN-FAST scores of 0, indicating low risk for a true IgE-mediated reaction. Two patients tolerated DOC but requested that their allergy label remain unchanged. One patient had a reaction that an ID physician determined to be unrelated to DOC, as they had a similar presentation the day before receiving a PCN, but could not be de-labeled. One patient developed a mild IgE-mediated hypersensitivity of pruritus and urticaria requiring diphenhydramine and steroids for resolution, although there was no evidence of a serious IgE-mediated response. This resulted in a 4% (one patient) reaction rate, which is percentage-wise higher than reported in PALACE (0.5% reaction rate). The PALACE trial included 187 patients in the intervention group compared to the 25 patients who underwent a challenge in our study, limiting the statistical interpretation between reaction rates given our small sample size [12]. These findings add to the literature supporting the safety and tolerability of DOC but also underscore how the implementation of pre-approved protocols and provider oversight is instrumental for programmatic de-labeling in a safe fashion.

We addressed an alternative way to impact future care for patients that could not have de-labeling or reconciliation performed by updating the allergy field with antibiotic agents that the patient could tolerate now and in the future. After de-labeling or allergy field updating, providers were made aware of these results to facilitate de-escalation to more narrow agents the patient could tolerate if antibiotics needed to be continued. In the FAR cohort, 36% of patients had their antibiotic regimen modified, which resulted in 78 days (28%) of therapy saved from either intravenous or broad-spectrum agents. In the screened cohort, only one more patient was de-labeled than the FAR cohort, but there were 49 more days of antibiotics saved. This highlights that communicated updates to the allergy field, even when patients do not undergo DOC, may have a positive impact on de-escalation and antibiotic optimization. One opportunity to improve our reach would be to assess patients earlier in their hospital stay. There was a median of 4 days between admission and FAR in this study, and only nine patients were assessed in the Emergency Department (ED). Shortening this time frame with prioritization of ED patients or expansion of PADLS hours would lead to earlier antibiotic optimization, including the benefits that come with it. The overall benefits of de-escalation are less quantifiable than other outcomes; however, these changes could reduce the risk of line-associated complications, improve antibiotic stewardship, potentially mitigate resistance, and decrease length of stay [4,7,21,22,23].

This study was able to show that PADLS could result in cost-savings for our institution. Sousa-Pinto et al. previously conducted an economic evaluation assessing direct medical costs associated with DOC and allergy de-labeling, incorporating variables such as patient characteristics, length of stay, readmission rates, adverse reaction probabilities, drug costs, and allergy testing expenses [20]. Their inpatient models estimated a savings of $1792 per DOC and de-labeling [20]. Applying these numbers to the 21 patients who were successfully challenged and de-labeled resulted in an estimated $37,632 saved over just 48 h of pharmacist time—approximately six workdays. When extrapolated to a standard 20-day work month, projected savings could reach $125,440. Even using more conservative estimates, such as those from Dong et al., who calculated $81 in drug cost savings per de-labeling, our program could still yield $2268 over six workdays, or approximately $98,280 annually [24]. While these cost-savings are estimates, they showcase the financial viability of a pharmacist-led allergy de-labeling initiative and indicate that further expansion of PADLS would be fruitful in ensuring equitable access to allergy assessment services and the generation of additional cost savings.

There are several limitations to this study that may have influenced the results. The retrospective, descriptive aspect of this study limits its generalizability to other institutions or comparison to other programs. PCN allergies were considered de-labeled if removed immediately following PADLS intervention. Instances where the allergy was subsequently relabeled into the EMR were not captured. Further steps to ensure longevity of de-labeling would include routine use of wallet cards affirming the results of the de-labeling, contacting other clinician offices that may have the allergy on file and advising them to remove it, and re-printing hospital bracelets while admitted to reflect the change in allergy status. With more dedicated time to PADLS, long-term de-labeling efforts such as these could be feasible. Patient selection for screening was subject to the discretion of the infectious diseases pharmacist, introducing potential bias towards individuals perceived as more likely to tolerate DOC, possess low PEN-FAST scores, and/or derive the greatest benefit from de-labeling. Antibiotic adjustments cannot all be attributed to PADLS or allergy field updating, as this was not documented at the time of adjustment, possibly confounding our results. Cost savings were estimated using fixed values from external sources, limiting the ability to account for institution-specific factors such as reductions in readmissions, IV line complications, adverse drug events, or length of stay. Additionally, despite education efforts and the Pharmacy & Therapeutics Committee approval of DOT and PST protocols, there remained limited awareness of de-labeling and PADLS efforts. Reconciliation efforts required real-time education to address nursing, patient, and provider hesitancy. Future programs should consider aligning champions or key stakeholders for primary provider groups early in the PADLS process.

## 5. Conclusions

This study demonstrates that an inpatient pharmacist-driven PCN allergy de-labeling service can improve antimicrobial stewardship, optimize antibiotic selection, and may be able to generate cost savings. The PADLS initiative successfully de-labeled most assessed patients, updated allergy documentation to guide future prescribing, and reduced unnecessary broad-spectrum and IV antibiotic use—saving 78 days of therapy and an estimated $37,632 in just 48 h of pharmacist time. When scaled, the program offers a replicable model for pharmacists to enhance patient safety and improve care efficiency.

## Figures and Tables

**Table 1:** PEN-FAST Penicillin allergy clinical decision rule.

Question			Response	Points
Five years or less since reaction?			Yes/unknown	+2
			No	0
Anaphylaxis or angioedema or severe cutaneous adverse reaction?			Yes	+2
			No	0
Treatment required for reaction?			Yes/unknown	+1
			No	0
Final Score	0 Points DOC offered	1–2 Points DOC offered	3 Points PST + DOC offered	4–5 Points Nothing offered

Adapted from *JAMA Intern Med.* 2023; 183(9): 944–952. [12]

**Table 2:** Baseline demographics.

		Screened (n = 63)	FAR (n = 32)
Age, median years (IQR)		63 (53, 73)	58 (48, 75)
Race, n (%)			
	Caucasian	46 (73)	23 (71.8)
	African American	9 (14.3)	4 (12.5)
	Hispanic	1 (1.6)	0 (0)
	Other	7 (11.1)	5 (15.6)
LOS, median days (IQR)		7 (3.5, 14)	6 (3, 15)
Days to assessment, median (IQR)		3 (2, 7)	3 (1, 4)
Assessment location, n (%)			
	Wards	45 (71.4)	24 (75)
	ICU	9 (14.3)	2 (6.3)
	ED	9 (14.3)	6 (18.7)
ID consult, n (%)		20 (31.7)	9 (28.1)
Types of infection, n (%)			
	Pneumonia	15 (23.8)	11 (34.3)
	SSTI	14 (22.2)	8 (25)
	UTI	13 (20.6)	4 (12.5)
	IAI	6 (9.5)	4 (12.5)
	BSI	4 (6.4)	1 (3.1)
	Bone/joint	1 (1.6)	1 (3.1)
	>1 infection	4 (6.3)	1 (3.1)
	Other	6 (9.6)	2 (6.2)

LOS: Length of Stay; FAR: Full Allergy Reconciliation; ICU: Intensive Care Unit; ED: Emergency Department; ID: Infectious Diseases; SSTI: Skin and Soft Tissue Infection; UTI: Urinary Tract Infection; IAI: Intra-Abdominal Infection; BSI: Bloodstream Infection.

**Table 3:** PEN-FAST assessments and challenges.

		FAR (n = 32)
PEN-FAST score, median (IQR)		0 (0, 1)
Patient-reported reaction, n (%)		
	Mild—Moderate *	14 (43.8)
	Severe **	4 (12.5)
	Delayed	0 (0)
	>1 reaction	2 (6.3)
	Intolerance ***	2 (6.3)
	Unknown	10 (31.3)
When did the allergy occur? n (%)		
	<5 years	1 (3.1)
	5–10 years	0 (0)
	11–20 years	2 (6.3)
	21–40 years	6 (18.8)
	>40 years	21 (65.6)
	Unknown	2 (6.3)
Did the allergy cause a hospitalization? n (%)		
	No	18 (56.3)
	Yes	12 (37.4)
	Unknown	2 (6.3)
Did the patient receive treatment? n (%)		
	No	21 (65.6)
	Yes	3 (9.4)
	Unknown	8 (25)
PCN challenge method? n (%)		
	Directly de-labeled	7 (21.9)
	1-step	24 (75)
	2-step	1 (3.1)
	Skin test	0 (0)
Did patients tolerate the challenge?		
	No	1 (4)
	Yes	24 (96)

*—itching, localized rash, palpitations, hives/blistering; **—shortness of breath, swelling of lips/tongue/throat, skin sloughing, fainting/collapsing; ***—gastrointestinal upset, nausea, vomiting, headaches.

**Table 4:** Outcomes.

	Screened (n = 63)	FAR (n = 32)
Primary Outcome		
PCN allergy de-labeled, n (%)	29 (46)	28 (87.5)
Secondary Outcomes		
PCN allergy field updated, n (%)	44 (69.8)	31 (96.9)
Antibiotic adjustment, n (%)	20 (31.7)	12 (37.5)
Total antibiotic duration of therapy, days	538	280
Total duration of therapy after adjustment, days	127	78
% of days adjusted	23.6%	27.8%
Cost Analysis		
Total cost savings for challenge + de-labeling ($1792/case)	---	$37,632

## Data Availability

The data presented in this study are available on request from the corresponding author due to institutional privacy restrictions.

## Abbreviations

The following abbreviations are used in this manuscript:

DOC	Direct Oral Challenge
ED	Emergency Department
EMR	Electronic Medical Record
FAR	Full Allergy Reconciliation
IRB	Institutional Review Board
MDROs	Multi-drug-Resistant Organisms
PADLS	Penicillin Allergy De-Labeling Service
PCN	Penicillin
PST	Penicillin Skin Testing
SOB	Shortness of Breath
SSTI	Skin and Soft Tissue Infection
TGH	Tampa General Hospital
UTI	Urinary Tract Infection

## References

[1] Khan D.A., Banerji A., Blumenthal K.G., Phillips E.J., Solensky R., White A.A., Bernstein J.A., Chu D.K., Ellis A.K., Golden D.B. (2022). Drug allergy: A 2022 practice parameter update. J. Allergy Clin. Immunol..

[2] Hawn M.T., Richman J.S., Vick C.C., Deierhoi R.J., Graham L.A., Henderson W.G., Itani K.M.F. (2013). Timing of surgical antibiotic prophylaxis and the risk of surgical site infection. JAMA Surg..

[3] Blumenthal K.G., Ryan E.E., Li Y., Lee H., Kuhlen J.L., Shenoy E.S. (2018). The Impact of a Reported Penicillin Allergy on Surgical Site Infection Risk. Clin. Infect. Dis..

[4] Blumenthal K.G., Kuper K., Schulz L.T., Bhowmick T., Postelnick M., Lee F., Walensky R.P. (2020). Association Between Penicillin Allergy Documentation and Antibiotic Use. JAMA Intern. Med..

[5] Steenvoorden L., Bjoernestad E.O., Kvesetmoen T.A., Gulsvik A.K. (2021). De-labelling penicillin allergy in acutely hospitalized patients: A pilot study. BMC Infect. Dis..

[6] Cooper L., Harbour J., Sneddon J., Seaton R.A. (2021). Safety and efficacy of de-labelling penicillin allergy in adults using direct oral challenge: A systematic review. JAC Antimicrob. Resist..

[7] Shenoy E.S., Macy E., Rowe T., Blumenthal K.G. (2019). Evaluation and Management of Penicillin Allergy: A Review. JAMA.

[8] Trubiano J.A., Vogrin S., Chua K.Y.L., Bourke J., Yun J., Douglas A., Stone C.A., Yu R., Groenendijk L., Holmes N.E. (2020). Development and Validation of a Penicillin Allergy Clinical Decision Rule. JAMA Intern. Med..

[9] Copaescu A.M., Vogrin S., Shand G., Ben-Shoshan M., Trubiano J.A. (2022). Validation of the PENFAST Score in a Pediatric Population. JAMA Netw. Open.

[10] Cox F., Vogrin S., Sullivan R.P., Stone C., Koo G., Phillips E., Li J., Fernando S.L., Al Gassim M., Mitri E. (2025). Development and validation of a cephalosporin allergy clinical decision rule. J. Infect..

[11] Stehlin F., Vogrin S., Mitri E., Isabwe G.A.C., Trubiano J.A., Copaescu A. (2025). International Validation of the SULF-FAST Risk-Stratification Tool for Sulfonamide Antibiotic Allergy. JAMA Netw. Open.

[12] Copaescu A.M., Vogrin S., James F., Chua K.Y.L., Rose M.T., De Luca J., Waldron J., Awad A., Godsell J., Mitri E. (2023). Efficacy of a Clinical Decision Rule to Enable Direct Oral Challenge in Patients with Low-Risk Penicillin Allergy: The PALACE Randomized Clinical Trial. JAMA Intern. Med..

[13] Rose M.T., Holmes N.E., Eastwood G.M., Vogrin S., James F., De Luca J.F., Bellomo R., Warrillow S.J., Phung M., Barnes S.L. (2024). Oral challenge vs routine care to assess lowrisk penicillin allergy in critically ill hospital patients (ORACLE): A pilot safety and feasibility randomised controlled trial. Intensive Care Med..

[14] Mir A., Lanoue D., Zanichelli V., van Walraven C., Olynych T., Nott C., MacFadden D. (2024). Introduction of a penicillin allergy de-labelling program with direct oral challenge and its effects on utilization of beta-lactam antimicrobials: A multicenter retrospective parallel cohort study. Allergy Asthma Clin. Immunol..

[15] Mann J., Cox V., Gorman S., Calissi P. (2024). Barriers to and Facilitators of Delabelling of Antimicrobial Allergies: A Qualitative Meta-synthesis. Can. J. Hosp. Pharm..

[16] Mahmud M., Kokoy S.R., Stollings J.L., McCoy A.B., Koo G., Dear M.L., Rice T.W., Phillips E.J., Stone C.A. (2025). Impact of a Pharmacist Driven Penicillin Allergy De-labeling Protocol on Rates of Reported Allergy in the Intensive Care Unit. Hosp. Pharm..

[17] Salazar González F., Iglesias Rodrigo M., Garreta Fontelles G., Mensa Vendrell M., Nicolás Picó J. (2026). Evaluation of penicillin allergy labeling in the emergency department: Retrospective study on the impact of a hospital pharmacist-led intervention algorithm. Farm. Hosp..

[18] Caturano B.N., Bjork L.L., Lichtenberger P.N., Temiño V.M. (2024). Proactive risk stratification and low-risk penicillin allergy delabeling by an antimicrobial stewardship pharmacist. Antimicrob. Steward. Healthc. Epidemiol..

[19] ALK-Abello, Inc (2013). PRE-PEN Package Insert.

[20] Sousa-Pinto B., Blumenthal K.G., Macy E., Pereira A.M., Azevedo L.F., Delgado L., Fonseca J.A. (2021). Penicillin Allergy Testing Is Cost-Saving: An Economic Evaluation Study. Clin. Infect. Dis..

[21] Tilanus A., Drusano G. (2023). Optimizing the Use of Beta-Lactam Antibiotics in Clinical Practice: A Test of Time. Open Forum Infect. Dis..

[22] Otani I.M., Tang M., Wang L., Anstey K.M., Hilts-Horeczko A., Li F., Le V.P., Lee M., Bystritsky R., Mulliken J.S. (2023). Impact of an Inpatient Allergy Guideline on β-Lactam and Alternative Antibiotic Use. J. Allergy Clin. Immunol. Pract..

[23] Alm R.A., Lahiri S.D. (2020). Narrow-Spectrum Antibacterial Agents-Benefits and Challenges. Antibiotics.

[24] Dong Y., Zembles T.N., Nimmer M., Brousseau D.C., Vyles D. (2023). A potential cost savings analysis of a penicillin de-labeling program. Front. Allergy.

